# Abortion through Sympathy

**Published:** 1880-10

**Authors:** 


### ABORTION THROUGH SYMPATHY.

It is well known to veterinarians that among cows abortion prevails apparently through sympathy, becoming epidemic in herds. The following case, reported by a writer in the *British Medical Journal*, looks as if the same thing may occasionally occur in the human species. He writes:

"Some days since I was hurriedly sent for to see a woman, wife of a small tradesman, who was said to be violently flooding. On my arrival, to my surprise, I found *two* women in the one bed. The one for whom I was especially sent was said to be now easier, and free from flooding but that the other (her sister) was very bad, her "womb having come down" suddenly, while looking after her sister. Upon examination I found a three months' fœtus born, all but the head. I removed the child and placenta, which followed immediately, and turned to my other patient, and found that she, too, was miscarrying, and at about the same period of gestation."—*Medical and Surgical Reporter.*

